# A narrative review of the potential pharmacological influence and safety of ibuprofen on coronavirus disease 19 (COVID-19), ACE2, and the immune system: a dichotomy of expectation and reality

**DOI:** 10.1007/s10787-020-00745-z

**Published:** 2020-08-14

**Authors:** Lucinda Smart, Neil Fawkes, Paul Goggin, Graham Pennick, K. D. Rainsford, Bruce Charlesworth, Neil Shah

**Affiliations:** 1 Reckitt Benckiser Health Ltd, Dansom Lane, Hull, HU8 7DS UK; 2 Reckitt Benckiser Health Ltd, 103-105 Bath Road, Slough, SL1 3UH UK; 3 Biomedical Research Centre, Sheffield Hallam University, Sheffield, S1 1WB UK

**Keywords:** Ibuprofen, COVID-19, SARS-CoV-2, ACE2, Immune system, Safety

## Abstract

The coronavirus disease 19 (COVID-19) pandemic is currently the most acute healthcare challenge in the world. Despite growing knowledge of the nature of Severe Acute Respiratory Syndrome coronavirus-2 (SARS-CoV-2), treatment options are still poorly defined. The safety of non-steroidal anti-inflammatory drugs (NSAIDs), specifically ibuprofen, has been openly questioned without any supporting evidence or clarity over dose, duration, or temporality of administration. This has been further conflicted by the initiation of studies to assess the efficacy of ibuprofen in improving outcomes in severe COVID-19 patients. To clarify the scientific reality, a literature search was conducted alongside considerations of the pharmacological properties of ibuprofen in order to construct this narrative review. The literature suggests that double-blind, placebo-controlled study results must be reported and carefully analysed for safety and efficacy in patients with COVID-19 before any recommendations can be made regarding the use of ibuprofen in such patients. Limited studies have suggested: (i) no direct interactions between ibuprofen and SARS-CoV-2 and (ii) there is no evidence to suggest ibuprofen affects the regulation of angiotensin-converting-enzyme 2 (ACE2), the receptor for COVID-19, in human studies. Furthermore, in vitro studies suggest ibuprofen may facilitate cleavage of ACE2 from the membrane, preventing membrane-dependent viral entry into the cell, the clinical significance of which is uncertain. Additionally, in vitro evidence suggests that inhibition of the transcription factor nuclear factor-κB (NF-kB) by ibuprofen may have a role in reducing excess inflammation or cytokine release in COVID-19 patients. Finally, there is no evidence that ibuprofen will aggravate or increase the chance of infection of COVID-19.

## Introduction

The world is currently impacted by the pandemic spread of Coronavirus-disease-2019 (COVID-19) caused by the Severe Acute Respiratory Syndrome coronavirus-2 (SARS-CoV-2). Efforts to overcome the morbidity and mortality caused by this novel virus have been hampered by a lack of knowledge of the SARS-CoV-2 virus, particularly with regard to virulence, individual risk factors to host responses and appropriate treatment (Rokni et al. 2020). It is becoming clear that the increased incidence of life-threatening cases caused by SARS-CoV-2 are, at least in part, a consequence of a cytokine storm in some individuals. It is hypothesised that imbalances within the renin–angiotensin–aldosterone system (RAAS) and angiotensin-converting enzyme 2 (ACE2) play a role in facilitating host cell infection (Hoffmann et al. 2020) or the inflammatory responses triggered by the SARS-CoV-2 virus (Liu et al. 2020a).

Concerns regarding the use of ibuprofen originated after the French Health Ministry issued advice on the 14th March 2020 to avoid using non-steroidal anti-inflammatory drugs (NSAIDs) to treat symptoms of COVID-19 (Day 2020). This triggered a debate within social and mainstream media as well as the scientific community, further propagated by the suggestion that ibuprofen could upregulate ACE2 (Fang et al. 2020b). Global shortages of paracetamol that occurred during the pandemic (Newton et al. 2020) compounded the issue, leaving patients with limited choice for symptomatic relief treatments in the face of mixed safety messages and little or no symptomatic treatment options for individuals for whom paracetamol was contraindicated.

Clinical judgement should be based on balanced scientific opinion and the principles of evidence-based medicine (Akobeng 2005). The generation and analysis of evidence is essential to provide treatment recommendations, influence healthcare professionals’ opinions and inform patient’s self-care decisions during times of crisis. Public questioning of the safety of a drug with a lack of substantiation or clarity over the dose, duration, chronicity, or class effect, renders making informed safety recommendations impossible. This shifts the responsibility of decision-making to practitioners, and ultimately to an excessively cautious approach in the face of significant uncertainty.

This article provides a safety and efficacy assessment of the pharmacological actions of ibuprofen and reviews the literature associated with ibuprofen’s potential benefits and risks in relation to SARS-CoV-2 infection.

## RAAS and its role in inflammation

**Fig. 1:**
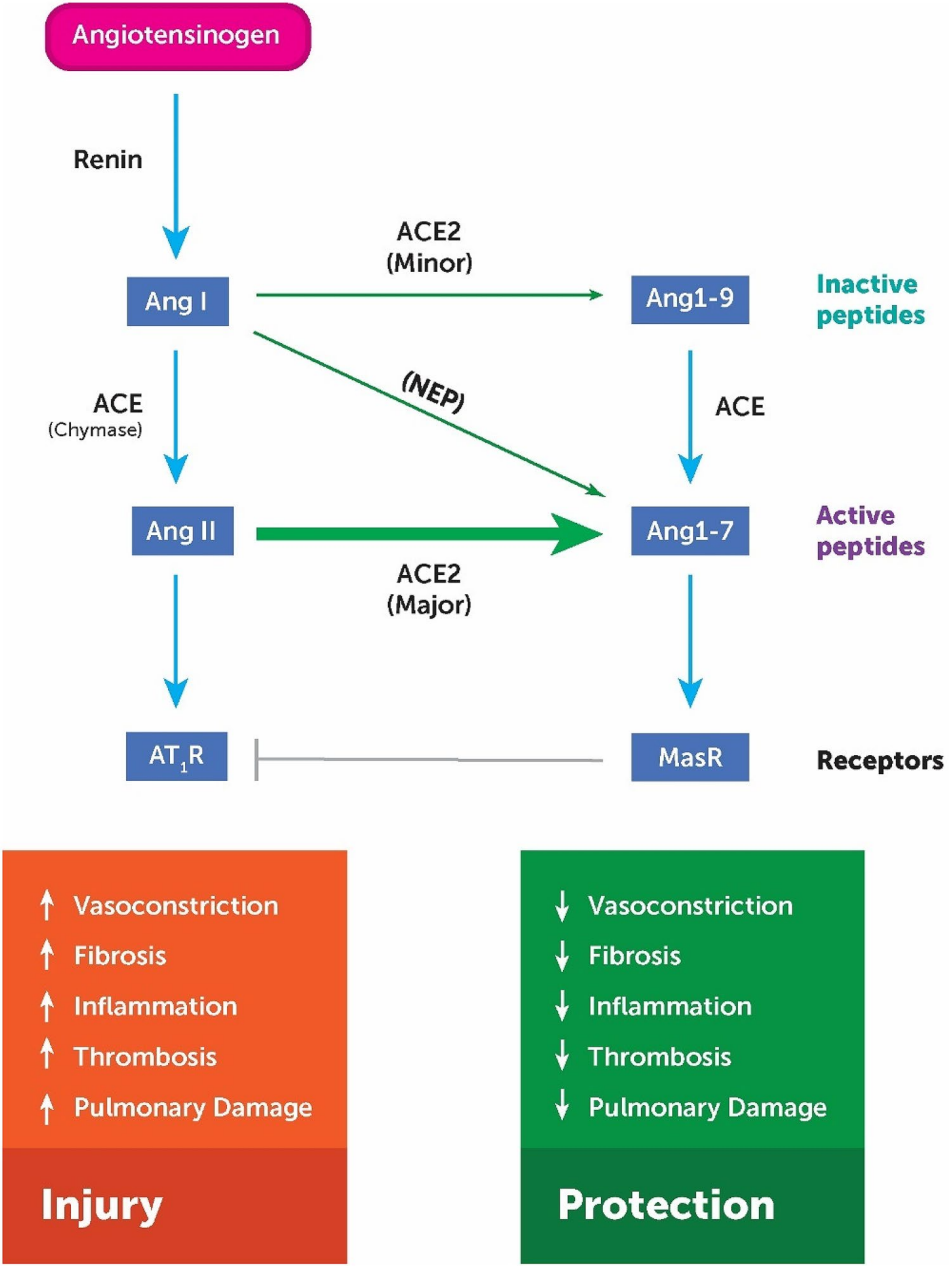
The balancing role of angiotensin-converting enzyme 2 (ACE2) in the renin–angiotensin–aldosterone system (RAAS). In a situation where ACE2 is downregulated, AngII levels rise and its concomitant binding on AT_1_R increases systemic inflammation via production of inflammatory mediators. *Ang* angiotensin, *NEP* neprilysin, *ACE* angiotensin-converting enzyme, *AT*_*1*_*R* angiotensin type 1 receptor, *AT*_*2*_*R* angiotensin type 2 receptor, *MasR* Mas receptor

The renin–angiotensin–aldosterone system (RAAS) is a complex system of enzymes, vasoactive peptides, and hormones which controls numerous functions in multiple organs. The role of the RAAS expands beyond the homeostatic regulation of blood pressure, electrolyte excretion, and fluid balance (Muñoz-Durango et al. 2016) and is now also considered to play an important role in the regulation of inflammation (Parajuli et al. 2014). The control mechanisms involved in the homeostatic regulation of inflammatory signalling pathways within the RAAS are shown in Fig. 1. The ACE2 axis forms a counter-regulatory pathway, opposing the pro-inflammatory ACE axis and its downstream effectors such as the inflammatory cytokine tumour necrosis factor alpha (TNFα) (Simoes e Silva et al. 2013).

Disruption to the homeostatic control mechanisms regulating these inflammatory pathways can contribute to the pathogenesis of disease such as cardiac hypertrophy, pulmonary hypertension, glomerulonephritis, lung injury, sepsis, and acute pancreatitis (Tikellis and Thomas 2012; Zisman 2005). Imbalances within the RAAS (such as an increased ACE:ACE2 ratio) favouring an increase in the active peptide angiotensin II (Ang II), result in amplification of the angiotensin II type I receptor (AT_1_R) expression and activity (Elton and Martin 2007). This includes surges of downstream inflammatory mediators, such as NF-κB, cyclooxygenase 2 (COX2), and mitogen-activated protein kinase (MAPK); as well as cytokine production such as interleukin-6 (IL-6), TNFα, and transforming growth factor beta 1 (TGF-β1) (Suzuki et al. 2003). The deleterious inflammatory effects of homeostatic failure of the RAAS pathway are highlighted in ACE2-knockout mice, where increases of reactive oxygen species (ROS), inflammatory cytokines, and enhanced oxidative stress are observed (Alghamri et al. 2013).

The study of patients with chronic complex immune-mediated diseases, such as rheumatoid arthritis and lupus, helps to extrapolate these findings to humans. The observation of increased activity of ACE in pleural fluid, increased adverse cardiovascular outcomes (mediated by vascular inflammation), and reversibility with the administration of ACE inhibitors (ACEis) or angiotensin receptor blockers demonstrate that an imbalance of the RAAS does exist in cases of chronic inflammation (Teplitsky et al. 2006; Zisman 2005).

### Angiotensin-converting enzyme 2

**Fig. 2:**
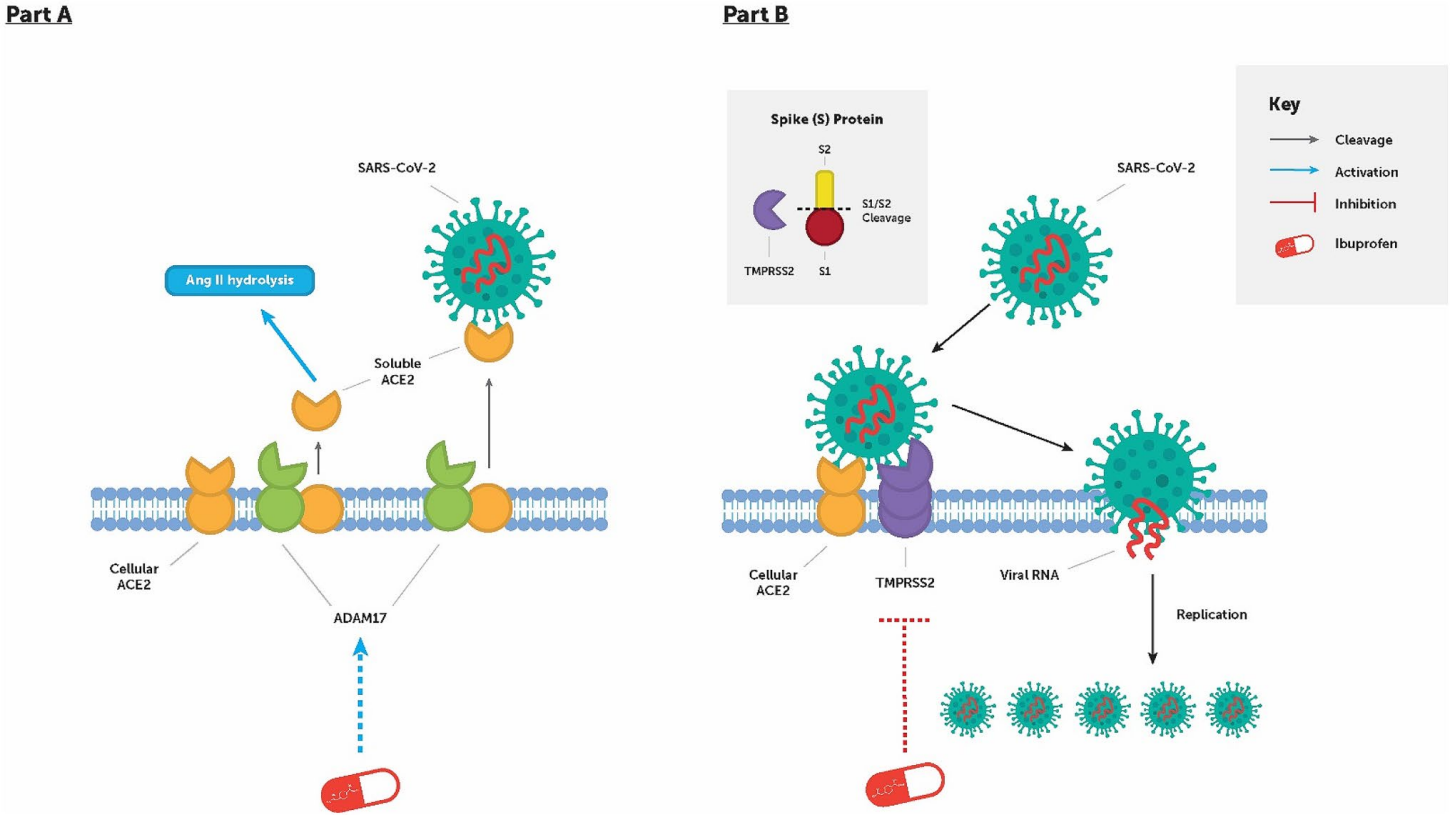
**a** The entry of severe acute respiratory syndrome coronavirus-2 (SARS-CoV-2) into cells is mediated by the binding of the viral spike (S) glycoprotein to membrane-bound angiotensin-converting enzyme 2 (ACE2). ACE2 levels on the plasma membrane are regulated by the metalloproteinase ADAM-17, which promotes shedding of ACE2 with its active N domain into the circulation. The cleaved portion of ACE2, which does not contain the membrane-bound domain necessary for viral entry into the cell, may act as a decoy for the viral spike proteins. It is hypothesised that ibuprofen may be able to activate ADAM-17 and thus prevent membrane-bound ACE2-dependent infection of the cell. **b** The enzyme transmembrane protein, serine 2 (encoded for by the TMPRSS2 gene) is necessary for the proteolytic cut at the S1/S2 site on the viral S glycoprotein for uptake into the cell and viral replication. It is hypothesised that ibuprofen may be able to downregulate expression of the enzyme, and thus prevent TMPRSS2-dependent viral entry into the cell

ACE2 has been identified as a binding site for the SARS-CoV-2 spike protein, which contains two distinct functional domains, termed S1 and S2, both of which are necessary for viral entry into a host cell (Fig. 2) (Hoffmann et al. 2020). ACE2 is a homologue of ACE and a key enzyme involved in the synthesis of bioactive components of the RAAS. It is a type 1 transmembrane protein (N-terminus extracellular, C-terminus intracellular) and it acts as a pleiotropic mono-carboxypeptidase responsible for production of angiotensin(1–7) via hydrolysing Ang II (Douglas et al. 2004). Despite the similarity, ACE2 differs from ACE both in its lack of inhibition by ACEis and substrate specificity (Donoghue et al. 2000).

The majority (approximately 83%) of ACE2-expressing cells are alveolar epithelial type II cells (AECII) (Zou et al. 2020) suggesting that SARS-CoV-2 has a high affinity for lung tissue and subsequent infection, although it is also expressed in many extrapulmonary tissues including heart, kidney, endothelium, and the small intestine (Crackower et al. 2002; Danilczyk et al. 2006; Hamming et al. 2004). Several factors are known to repress ACE2 activation, such as the presence of pro-inflammatory cytokines (Chen et al. 2020a), the action of aldosterone via a NADPH oxidase pathway (Keidar et al. 2005), and stimulation of AT_1_R (Igase et al. 2005).

ACE2 contains only a single active domain (N domain) rather than the two catalytic domains (N and C domains) of ACE (Towler et al. 2004). This is of significance when it is considered that ACE2 can be cleaved from the membrane of cells in which it is expressed (Epelman et al. 2008), mediated by the protease ADAM-17, which causes its release into a soluble form with a catalytically active soluble N ectodomain that circulates in the extracellular environment (Lambert et al. 2005). In normal health status, circulating ACE2 levels are low compared with membrane-bound levels (Rice et al. 2006). Interestingly, given that the circulating form of ACE2 lacks the membrane anchor used as the cell entry point for SARS-CoV-2, it has been presented as a potential therapeutic target for prevention of infection of the virus, via acting as a decoy for the viral S-protein required for entry into its host’s cells (Batlle et al. 2020).

Generally speaking, ACE2 expression is low in good health, but levels are seen to rise in a number of pathologies (Lew et al. 2008) which incidentally are most grossly associated with more severe courses of infection. For example, disorders such as diabetes, hypertension and cardiovascular disease are associated with imbalances in the RAAS (Zemlin and Wiese 2020); changes to expression levels are suggested to be compensatory rather than causal as the system shifts the balance in order to rescue damage and restore homeostasis. It is worth noting that variation exists in its expression and regulation, depending on the organ and tissue type in question. Genetic variants in the gene encoding ACE2 have the potential to affect ACE2 levels in the human body: the Leeds Family Study, a well-characterised population of 89 healthy probands and their first-, second-, or third-degree relatives, elucidated that up to 67% of the phenotypic variation in circulating ACE2 levels could be accounted for by genetic factors, ascertained by measuring ACE, ACE2, and neutral endopeptidase (NEP) activities in plasma (Rice et al. 2006). The coding for ACE2 is located on the X-chromosome (Komatsu et al. 2002), which is hypothesised to afford greater protection to females than males in situations of severe illness, such as acute respiratory distress syndrome (ARDS) (Garami 2020), via ‘double dose’ gene effects. ARDS is a further clinical manifestation of severe viral infection.

### RAAS, ACE2 and COVID-19

ACE2 is not the only factor necessary for viral entry into the cell. As previously described, the spike (S) protein of SARS-CoV-2 contains two distinct functional domains, S1 and S2, of which both are necessary for viral entry into a host cell. S1 is responsible for the first stages of viral entry and contains the receptor-binding domain. S2 acts in the later-stage fusion of the cell and viral membranes and contains amino acid sequences necessary for continuing infiltration. For fusion to take place, the S-protein is cleaved by cellular proteases; this step is critical, as it allows for the fusion sequences to be exposed (Belouzard et al. 2009; Walls et al. 2020).

Such cleavage of membrane-bound ACE2 is thought to be facilitated by transmembrane protease serine 2 (TMPRSS2), which has been demonstrated to be essential for early entry and viral spread of SARS-CoV-2, through splitting the spike protein and establishing further penetration changes (Fig. 2) (Iwata-Yoshikawa et al. 2019). An Italian cohort study found no significant evidence that ACE2 is associated with disease severity/sex bias in the Italian population; rather, their analysis suggested a role for TMPRSS2 variants and expression levels in modulating SARS-CoV-2 severity (Asselta et al. 2020).

SARS-CoV-2 is capable of downregulating the levels of ACE2 receptors upon the surface of infected cells (Zhang et al. 2020a). Binding to ACE2 stimulates clathrin-dependent endocytosis of both the virus and the receptor; furthermore, binding of the spike protein also induces ADAM-17 activity, in turn reducing the amount of ACE2 expressed on the cell surface (Clarke and Turner 2012). There are two likely implications of a downregulation of ACE2: (1) that circulating levels of Ang II will increase in the absence of an enzyme capable of cleaving it; (2) protective levels of Ang(1–7) will decrease, reducing its conferred protective and anti-inflammatory effects and tipping the balance in favour an inflammatory state.

Given the observation that system-wide imbalances in the ACE:ACE2 ratio favouring the pro-inflammatory ACE axis are commonly observed in certain disease states commonly comorbid in severe COVID-19, it could be hypothesised that immune dysregulation of the RAAS and associated increases in pro-inflammatory mediators in the lungs may play a central role in the pathophysiology of SARS-CoV-2, such as induction of acute lung injury (ALI) or ARDS—both of which are notable causes of death in critically ill patients suffering from COVID-19 (Wu et al. 2020). This is supported by the fact that SARS-CoV-2 infection further exacerbates the imbalance, by downregulating ACE2 upon infection (Zhang et al. 2020a). Furthermore, age is an independent risk factor for mortality in patients admitted with COVID-19 (Ruan et al. 2020), potentially correlating with the observation that ACE2 expression reduces with age in rats (Xie et al. 2006). The data also suggest a disproportionate rate of disease occurrence, severity, and worse outcomes in males compared to females during this pandemic (Jin et al. 2020). These associations might be interpreted to indicate that low ACE2 during times of health is appropriate, but the ability to regulate overactive RAAS or the RAAS–SCoV axis during illness is, at least in part, dependent on ACE2. For instance, one study identified a higher expression of the lung-protective AT_2_ receptor among women, which could explain the disproportionately lower mortality in female COVID-19 subjects (Hilliard et al. 2012). Furthermore, as previously discussed, women and children are seen to have elevated levels of circulating ACE2 in comparison to men and the elderly (Ciaglia et al. 2020).

An upregulation of ACE2 has been suggested to offer pulmono-protective effects against the lung damage triggered by the virus, by reducing Ang II binding on AT_1_R and thus reducing production of downstream damaging effectors such as TGF-β1 (Kuba et al. 2005). Therefore, it is not fully understood whether higher levels of ACE2 might paradoxically confer increased protection against damage mediated by the virus. It is worth noting that intraperitoneal injections of recombinant human ACE2 protein at a dose of 0.1 mg kg^−1^ into mice has conferred protection against severe ALI, as measured by percentage change in lung elastance induced by acid aspiration (which mimics human ALI/ARDS, frequently caused by aspiration of gastric contents) (Imai et al. 2005).

**Fig. 3:**
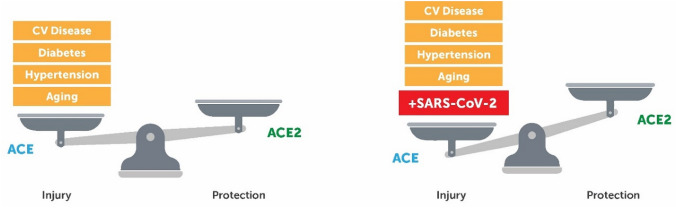
Balance in favour of the ACE–Ang II–AT_1_R axis over the ACE2–Ang(1–7)–Mas axis in the development of or injury in certain disease states. Infection with severe acute respiratory coronavirus 2 (SARS-CoV-2) only serves to further reduce levels of angiotensin-converting enzyme 2 (ACE2), pushing the balance further in the direction of injury for those with certain comorbidities. CV indicates cardiovascular disease

Such protective functions of ACE2 in the body may also help to explain why patients with existing comorbidities (such as diabetes, hypertension and cardiovascular disease) are most grossly associated with more severe courses of infection with SARS-CoV-2. When it is considered that individuals with these comorbidities have a lower level of ACE2 at baseline compared to their healthy counterparts, infection further exacerbates this imbalance in ACE2 compared to ACE, leading to a heightened predisposition to inflammatory damage (Fig. 3) (Oudit et al. 2009). Although small and with limited capacity for repeat, a cohort study of 12 SARS-CoV-2 patients demonstrated that circulating Ang II levels were markedly elevated compared to healthy controls, which was linearly correlated with viral load (*P* = 0.035) (Liu et al. 2020b), indicating that such downregulation of ACE2 could facilitate the multi-organ damage associated with viral infection.

## RAAS, COVID-19 and ibuprofen

RAAS plays a particularly important role in the course of SARS-CoV-2 infection. This interaction appears to be multifactorial, through facilitation of viral entry into the host cell and modulation of the inflammatory response observed in reaction to the virus. Equally, ibuprofen has multiple modes of action that underpin its pharmacology, which are dependent on its pharmacokinetics.

### Antiviral properties

Ibuprofen is administered as a racemic mixture: the R(−)-enantiomer undergoes extensive interconversion to the S(+)-enantiomer in vivo. The S(+)-enantiomer is considered to be the more pharmacologically active enantiomer and is capable of inhibiting cyclooxygenase (COX), a key enzyme responsible for inflammation and pain, at clinically relevant concentrations. On reviewing the literature, no studies were found to indicate that either the R(−) or S(+)‐enantiomers would structurally interact with the virus, specifically the spike proteins, which are essential to anchor the virus to the cells surface. Again, no studies were found within the literature to demonstrate that either enantiomer would directly interact with either receptors present on ACE2 or TMPRSS2 providing competition or affecting affinity of the SARS-CoV-2 virions.

Ibuprofen is not considered to be an antiviral agent, however a potential indirect relationship between ibuprofen and ACE2 is described in vivo using streptozotocin-induced diabetic rats which were fed ibuprofen (40 mg/kg—with allometric scaling, human equivalent dose equates to 450 mg for a 70 kg person) once daily by gavage for 8 weeks (Qiao et al. 2015). The results were evaluated by assessment of cardiac fibrosis and evaluation of TGF-β1 and the mammalian target of rapamycin (mTOR), both major components of the RAAS. This was a small study; however, a beneficial outcome of ibuprofen, which ameliorated the development of cardiac fibrosis in the diabetic group, was observed—an effect that was hypothesised to be the result of normalisation of the ACE:ACE2 ratio in the diabetic mice. The mode of enhancement of the ACE2/Ang(1–7)/MasR axis by ibuprofen likely occurred via its direct inhibitory effects on the downstream pro-inflammatory mediators (e.g. NF-kB, COX2 & TGF-β1) of the ACE/Ang II/AT_1_R axis. In support of this, the study also suggests that the normalisation of the ACE:ACE2 ratio is likely to be an indirect pharmacodynamic effect: a consequence of the restoration of the inflammatory imbalance caused by diabetic dysregulation of the RAAS in favour of ACE and a pro-inflammatory state. However, no effect was seen in the control group which did not have induced diabetes. In the context of COVID-19, extrapolation of this result into healthy humans should be undertaken with caution, given the dosage regime in the study (40 mg/kg daily for 8 weeks) is not comparable to a standard maximum daily OTC dose of 1200 mg of ibuprofen.

It could be hypothesised that ibuprofen may also indirectly impact the cleavage of ACE2 from the cell membrane, thus limiting viral infectivity, given that the circulating form of ACE2 lacks the membrane anchor used as the cell entry point for SARS-CoV-2. The suggested mechanism mediating such effect is that ibuprofen’s insertion into the lipid bilayer of the cell membrane (Rojas-Valencia et al. 2018) facilitates activation of the NADPH oxidase complex and thus the release of superoxide anions into the extracellular milieu, which in turn generate an oxidative attack on the prodomain thiol groups (which mask the active site of ADAM-17), causing their removal (Diaz-Gonzalez and Sanchez-Madrid 2015) and thus activation to process ACE2 cleavage. Given the lack of evidence of a direct pharmacodynamic interaction with ACE2 or its mediators, it is not understood that ibuprofen leads to an overexpression of ACE2 and thus viral entry, as has been implied (Fang et al. 2020b). It is more feasible to suggest that ibuprofen provides either no benefit or a possible protective effect, either through the attenuation of the enhanced inflammatory states observed in disease states identified to have worse outcomes in COVID-19, or through cleavage of membrane ACE2 to soluble ACE2, which is being considered as a plausible therapeutic opportunity (Batlle et al. 2020).

Furthermore, ibuprofen could impact viral entry via indirect interaction with TMPRSS2. TMPRSS2 is androgen-regulated (Lin et al. 1999); a randomised double-blinded placebo-controlled trial with an intervention of ibuprofen taken at a dose of 1200 mg daily for 6 weeks demonstrated that ibuprofen reduced the production of the androgen testosterone in a dose-dependent manner, likely through transcriptional repression (Kristensen et al. 2018). In vitro studies support this mechanism through the inhibition of testosterone glucuronidation in human liver microsomes via inhibition of the enzymes that facilitate the process (such as the UDP-glucuronosyltransferase, UGT2B15) (Sten et al. 2009). The relevance of this evidence in the context of COVID-19 infection is unclear. It could be hypothesised that ibuprofen could present a benefit in males through downregulation of TMPRSS2 via induced hypogonadism, however the extent of downregulation is still largely unknown. Any benefit would also need to be weighed up against any adverse effects of inducing a compensated hypogonadism and the relevance of acute symptomatic OTC dosing, given that a minimum of 14 days would be needed to see any potential effects.

### Modulation of the immune response

Like other respiratory viruses (Ramos and Fernandez-Sesma 2015), SARS-CoV-2 infection triggers a local immune response, recruiting macrophages and monocytes that respond to the infection, release cytokines, and prime adaptive T and B cell immune responses. In most cases, this response capably resolves the infection and clears the virus. However, in other cases, a dysfunctional immune response occurs which can cause severe lung and/or systemic injury. This is highlighted by the finding that in the blood of patients infected with SARS-CoV-2, there was a marked increase in interleukin 1β (IL-1β), interferon γ (IFN-γ), interferon-inducible protein 10 (IP-10), and monocyte chemoattractant protein 1 (MCP-1), as well as IL-4 and IL-10 when compared to that of SARS patients (Zhang et al. 2020b). It has therefore been hypothesised that excessive inflammation and an active cytokine storm contribute significantly to the pathogenesis of COVID-19.

The transcription factor NF-kB controls the expression of genes such as COX2, amongst a whole host of other downstream inflammatory mediators, and has multiple implications in the pathogenesis of SARS-CoV-2 infection. Firstly, NF-kB inhibition is seen to prevent upregulation of AT_1_R in Zucker diabetic fatty rats (Luo et al. 2015), indicating its capability to promote expression of the AT_1_R receptor and thus downstream inflammatory mediators. Secondly, NF-kB is witnessed to be utilised by the SARS-CoV virus to upregulate miRNA-200c-3p, which is capable of downregulating ACE2 via binding the 3′-untranslated region of ACE2 (Liu et al. 2017).

NSAIDs, and thus ibuprofen, are known to inhibit NF-kB activity during inflammatory responses: both the R(−)‐enantiomer and the S(+)‐enantiomer inhibited the activation of NF‐κB in response to stimulation by prostaglandin E_2_ (PGE_2_), a key mediator of inflammation, in Human Jurkat T‐lymphocytes (Scheuren et al. 1998). Normalising the levels of NF-kB may allow for the prevention of upregulation of AT_1_R and downregulation of ACE2 when it is over-expressed in the presence of inflammation or viral infection. However, the clinical meaningfulness of the known effects of ibuprofen on cytokines (be it pro- or anti-inflammatory) are currently unknown in COVID-19 patients: the dose, timings of dose, severity of condition and the state of an individual’s immune response are all potential determining factors and should be thoroughly assessed before conclusions can be drawn.

Interleukin-6 (IL-6), downstream of NF-kB, is over-produced in COVID-19 patients (Chen et al. 2020b). Interestingly, IL-6 leads to a relevant increase in cathepsin L, a lysosomal endopeptidase enzyme that can contribute to the internalisation and activation of SARS-CoV-2 (Abassi et al. 2020). Consequently, there are clinical trials involving anti-IL-6 therapies for the treatment of COVID-19 that are actively recruiting. Ibuprofen has been found to reduce IL-6 in human synovial fluid (Gallelli et al. 2013) and in sputum (Chmiel et al. 2015); therefore, it will be important to report the use of ibuprofen (amongst other NSAIDs) in clinical studies of COVID-19 in order to better understand what impact it may have on cytokine production.

Administration of anti-inflammatory molecules provides therapeutic opportunities to modulate such a response. The anti-inflammatory actions of ibuprofen are well established and in properly selected patients may be a reasonable approach to help reduce inflammatory damage related to the “cytokine storm”. For example, ibuprofen is documented to variously affect the production of the pro-inflammatory cytokines (IL‐1β, TNFα), as well as some intracellular signalling pathways in inflammatory cells (Rainsford 2015).

**Fig. 4:**
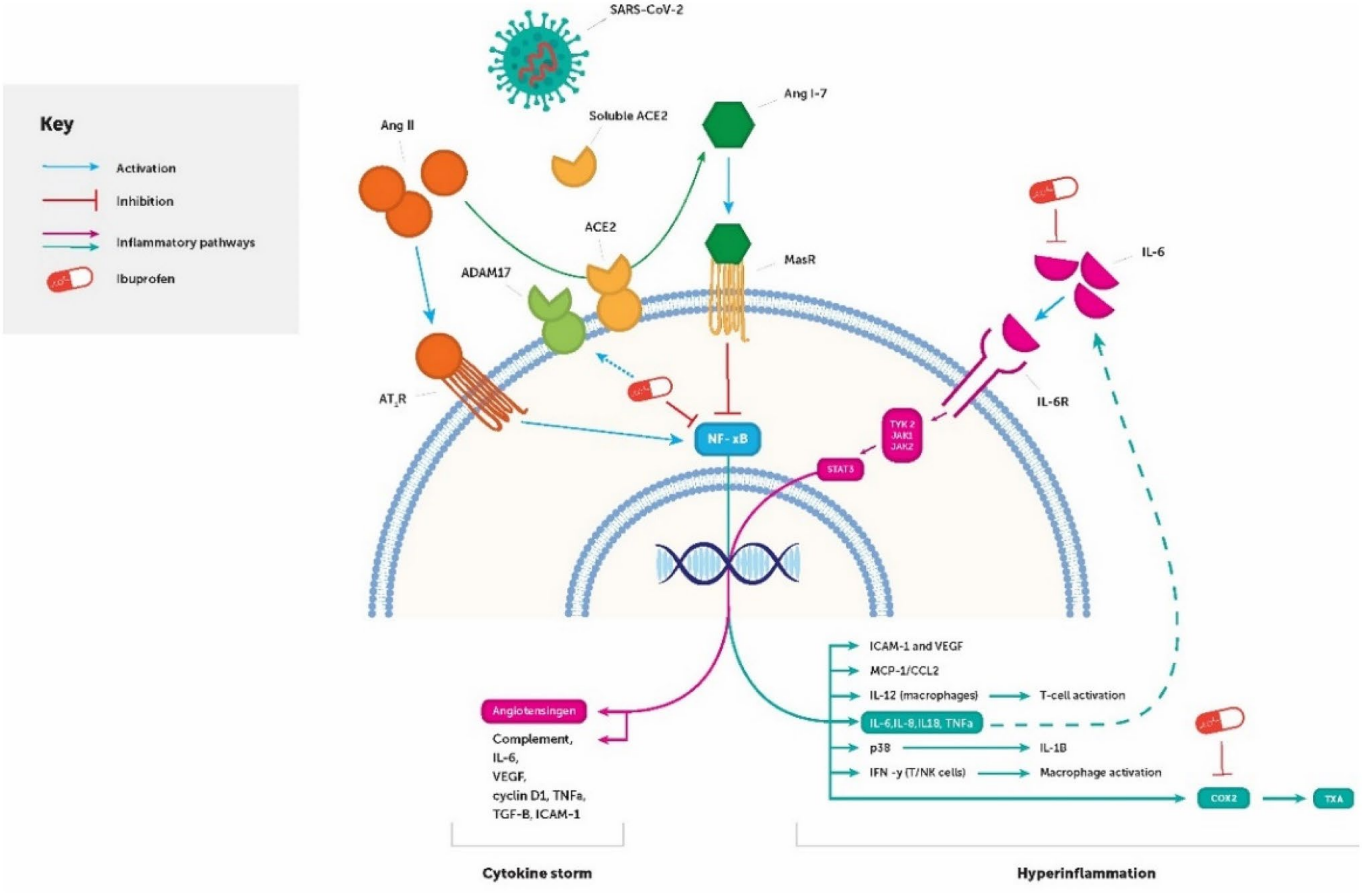
Interaction of severe acute respiratory coronavirus 2 (SARS-CoV-2) with the inflammatory system via downregulation of ACE2 and potential therapeutic effects of ibuprofen in reducing inflammation. In the presence of the virus, ACE2 is downregulated, thus preventing enzymatic hydrolysis of AngII into Ang(1–7). The resulting effect is there is increased production of the transcription factor NF-kB, which activates production of further inflammatory mediators. Ibuprofen has demonstrated ability to inhibit NF-kB, as well as other downstream inflammatory mediators such as interleukin-6, that seem to be upregulated upon viral infection. Furthermore, ibuprofen is hypothesised to activate ADAM-17 cleavage of ACE2 from the cell membrane, preventing viral entry into the cell. ACE = angiotensin-converting enzyme. *Ang* angiotensin, *ATR1* type 1 angiotensin II receptor, *AT2R* type 2 angiotensin II receptor, *COX2* cyclooxygenase 2, *ICAM* intercellular adhesion molecule, *IL* interleukin, *IFN* interferon, *JAK* Janus kinase, *MasR* Mas receptor, *NF-kB* nuclear factor kappa-light-chain-enhancer of activated B cells, *SARS-CoV-2* severe acute respiratory syndrome coronavirus 2, *STAT3* signal transducer and activator of transcription 3, *TGF* transforming growth factor, *TLR* toll-like receptor, *TNF* tumour necrosis factor, *TXA* thromboxane, *TYK* tyrosine kinase, *VEGF* vascular endothelial growth factor

In this capacity, it is plausible that anti-inflammatory therapies that reduce NF‐κB levels, such as non-acetylated salicylates (salsalate), as well as ibuprofen may be able to prevent the damage caused by unabated cytokine storms and hyperinflammatory states associated with COVID-19 (Fig. 4).

## Discussion

In response to the current SARS-CoV-2 outbreak, the collective, rapid output of research and literature around the world has been unprecedented, as scientists and academics attempt to understand the virus as the pandemic spreads. There has also been a proliferation in the availability of open-access journals or pre-print articles with little or no peer-review processes, which may lend themselves to become an important source of sensationalised news. The intent of this review article was to consider the salient and clinically relevant information within the literature in order to further understand the risk:benefit profile of the administration of OTC doses of ibuprofen in the context of COVID-19.

The literature contains strong evidence for the role of the RAAS, and in particular ACE2, in facilitating host cell infection, its downregulation thereof, and the consequential immune response triggered by the SARS-CoV-2 virus. We found no evidence in the literature to suggest a direct interaction between ibuprofen and ACE2 in humans. What is becoming increasingly clear is that severe and life-threatening cases of COVID-19 are, at least in part, a consequence of an excessive inflammatory response known as a “cytokine storm”. The specific components and triggers of the immune response in these patients are still not yet fully understood, but it is thought that such inflammatory mediators triggered by the virus may aberrantly destroy healthy cells (Tay et al. 2020). In addition to the interaction between SARS-CoV-2 and ACE2 in host cell infection, the literature also provides biological plausibility into how the RAAS could modulate the observed inflammatory response, recognising its physiological role as a key regulatory system of inflammation. The anti-inflammatory actions of ACE2 may also explain some of the independent risk factors identified in emerging observational data, such as age (declining ACE2 and an ageing immune system) (Xie et al. 2006) and comorbidities such as diabetes and cardiovascular disease (associated with a dysregulation of the RAAS in favour of ACE) (McFarlane et al. 2003). Emerging knowledge of the anti-inflammatory nature of ACE2 and the downstream effects of Ang(1–7) binding to MasR has called into question the protective effects that increased levels of ACE2 might afford an individual infected with SARS-CoV-2. It is certain that as more observational and epidemiological data are generated globally from infected patients, an enhanced understanding of the true situation surrounding ACE2 expression levels in the susceptibility to, and severity of, SARS-CoV-2 infection will be gained.

With fever and arthralgia being some of the most common symptoms displayed during the course of infection (Huang et al. 2020), the need for symptomatic relief and the ability to access appropriate self-medication is appreciable. Ibuprofen, aspirin and paracetamol are amongst the most widely used analgesic–antipyretic medicines in the world. In terms of low-dose, short-duration use in otherwise healthy adults, ibuprofen is noted to be the least toxic of the three, demonstrated by the fact that it is rarely associated with serious adverse events in low-risk patients (Derry et al. 2013; Rainsford 2012). This is attributable to the low dose–response curves for the anti‐inflammatory actions of ibuprofen, compared to the relatively high doses required for toxicity, thus giving this drug flexibility in its high therapeutic index, especially when compared with that of other NSAIDs and paracetamol (Rainsford 2012). The safety of ibuprofen use in SARS-CoV-2 infection has been questioned in a theoretical sense after demonstration of a potential to upregulate ACE2 expression in a singular animal model (Qiao et al. 2015). The foundation of this hypothesis is based on a single study in diabetic rats exposed to a daily dose of ibuprofen for 8 weeks (40 mg/kg, corresponding with 450 mg dose for a 70 kg person). The increase in ACE2 was shown in the heart only, and no distinction was made between membrane-bound ACE2 and soluble ACE2. The authors speculated that the ACE2 rise was due to inhibition of COX and/or activation of peroxisome proliferator-activated receptor γ without providing any evidence to support these claims. The citation of this finding in further literature (Fang et al. 2020b) postulated further unverified suggestions that increased expression of ACE2 would lead to enhanced susceptibility to viral infection and/or viral titres in cells, increasing the severity of the infection and leading to poorer prognoses. This review article provides a hypothesis on the underpinning mechanism which could explain the observed results or refute these positions.

Other speculative postulations against the use of NSAIDs, and ibuprofen in COVID-19, include suggestions that changes in the adaptive immune response caused by anti-inflammatory, antipyretic agents may dampen natural host responses that are necessary to fight viral infection or even mask signs of infection (Fang et al. 2020a). However, no evidence was found to support that this may be the case or define the level of risk. Data emerging from China have demonstrated that in almost all cases of severe infection, high levels of IL-6 and many other pro-inflammatory cytokines (predominantly downstream of NF-kB) and TNFα were detected. Ibuprofen has multiple modes of action that underlie its anti-inflammatory effects including, but not limited, to the downregulation of inflammatory pathways such as NF-kB and inhibition of pro-inflammatory cytokines IL-6 and TNFα. This suggests ibuprofen to be a safe and effective anti-inflammatory in the instance of SARS-CoV-2 infection in properly selected patients with low risk of NSAID-related complications. Further studies are warranted to establish the efficacy and mechanism of the control of inflammation in COVID-19 patients and associations with ACE2, in the context of ibuprofen and other drugs which may have similar modes of action. Ideally, clinical data will be generated in the form of the current gold standard requirements: mechanistic understanding will be gleaned from randomised, controlled clinical trials, which are designed to mitigate the protopathic bias that frequently confounds observational studies. However, it is acknowledged that with current restraints imposed by the pandemic, this data will be difficult to generate.

## Conclusion

This review explored the literature for any potential alteration of the risk/benefit profile in relation to SARS-CoV-2 infection. In conclusion, there are tenable arguments for both additional benefit and risk, however the evidence base is insufficient to allow clinically meaningful conclusions to be drawn. At this time, ibuprofen should continue to be used as normal, with no change in the warnings for practitioners and patients alike and according to the licensed indications and instructions for use, remaining in consideration as part of the symptomatic treatment options in COVID-19—advice supported by all international professional and regulatory bodies. This is further confirmed by the analysis of observational data acquired from patient registries of SARS-CoV-2-positive patients in (i) Israel, which demonstrated that ibuprofen use was not associated with worse clinical outcomes compared with paracetamol or no antipyretic (Rinott et al. 2020); (ii) Michigan, US, which demonstrated that NSAID use prior to admission was not associated with renal failure or increased mortality (Imam et al. 2020); and (iii) individuals with rheumatic diseases who demonstrated that exposure to NSAIDs was not associated with increased risk of hospitalisation (Gianfrancesco et al. 2020).

There is no current evidence to suggest a mechanism by which ibuprofen exacerbates, aggravates, or increases the chance of infection with SARS-CoV-2.
